# Extracellular CIRP dysregulates microglial efferocytosis in ischemic stroke via the TLR4/miR-155/MafB axis

**DOI:** 10.21203/rs.3.rs-7223452/v1

**Published:** 2025-07-31

**Authors:** Dmitriy Lapin, Dilara Aylar, Archna Sharma, Ping Wang

**Affiliations:** The Feinstein Institutes for Medical Research; The Feinstein Institutes for Medical Research; The Feinstein Institutes for Medical Research; The Feinstein Institutes for Medical Research

**Keywords:** Ischemic stroke, Neuronal death, Neuroinflammation, eCIRP, Microglia, TLR4/miR-155/MafB axis, Efferocytosis, C23 peptide, Therapeutics

## Abstract

**Background.:**

Ischemic stroke remains a leading cause of mortality and disability worldwide, but efforts to develop efficacious neuroprotective therapy face ongoing challenges. Efferocytosis, the phagocytic clearance of dying cells, by microglia is crucial for limiting neuroinflammation and promoting stroke resolution. Extracellular cold-inducible RNA-binding protein (eCIRP) is an inflammatory mediator which impairs macrophage bacterial phagocytosis in sepsis and radiation injury, but its role in microglial efferocytosis in ischemic stroke has not yet been studied.

**Results.:**

Using a transient middle cerebral artery occlusion (tMCAO) model of ischemic stroke, we demonstrate that eCIRP is released into the cerebrospinal fluid and microglial expression of crucial efferocytic receptor MerTK decreases in tMCAO mice. CIRP deficiency significantly improved neurological deficit, MerTK expression and microglial efferocytosis in tMCAO mice. Utilizing tMCAO, hippocampal injections, and primary microglia, we show that eCIRP induces pro-inflammatory micro-RNA 155 (miR-155) via TLR4, which suppresses its target pro-efferocytic transcription factor MAF bZIP (MafB), downregulating MerTK and microglial efferocytosis. Pharmacological blockade of eCIRP–TLR4 interaction using small peptide C23 attenuates miR-155 induction, restores MerTK expression, rescues microglial efferocytosis, and improves outcomes in tMCAO mice.

**Conclusion.:**

We show that eCIRP causes microglial efferocytic dysfunction in ischemic stroke via TLR4/miR-155/MafB axis. These findings uncover a previously unknown pathway through which eCIRP signaling impairs neuroprotective function in microglia and suggest that targeting eCIRP may promote functional recovery after stroke.

## Introduction

Stroke is the 2nd leading cause of death, and 3rd leading cause of combined death and disability worldwide [1, 2]. Of all stroke subtypes, ischemic stroke (IS) is the most common, making up 65% of all incident strokes [2]. Despite the massive global burden of IS, there has been a distinct lack of success in the development of novel efficacious therapies for IS [3]. During and after cessation of ischemia, brain cells release soluble pro-inflammatory molecules such as cytokines and damage-associated molecular patterns (DAMPs), producing a pro-inflammatory milieu [4]. Although the post-stroke inflammatory response is necessary for eventual tissue repair, the exaggerated acute phase of this response is known to amplify tissue damage and expand infarction, representing an opportunity for targeted pharmacologic intervention [5].

Cold-inducible RNA-binding protein (CIRP) is a small protein which is ubiquitously and constitutively expressed, canonically known to function as an RNA chaperone [6, 7]. We discovered that CIRP can be released extracellularly into circulation in sepsis and hemorrhagic shock [8]. Extracellular CIRP (eCIRP) then acts as a pro-inflammatory DAMP by binding to pattern recognition receptors toll-like receptor 4 (TLR4), triggering receptor expressed on myeloid cells-1 (TREM-1), and interleukin 6 receptor α (IL-6Rα) to induce deleterious inflammatory signaling pathways [8–11]. Recent studies in IS have demonstrated eCIRP’s injurious role, underscoring the need to improve our understanding of eCIRP dynamics in IS [12–16]. CIRP deficient (CIRP^−/−^) mice subjected to permanent middle cerebral artery occlusion (pMCAO) exhibit reduced microglial activation and smaller infarcts compared to control mice [12]. A recent study in a murine temporary middle cerebral artery occlusion (tMCAO) model of IS, showed that CIRP expression drives brain expression of pyroptosis-related proteins, augments pro-inflammatory polarization in microglia, and increases infarct volume, potentially via the NF-κB/NLRP3 pathway [15]. eCIRP also disrupts BBB integrity and promotes cerebral edema, a potentially deadly sequela of IS [15]. Human data corroborates eCIRP’s role as an important DAMP in IS. Serum eCIRP levels in patients correlate with clinical IS severity and outcomes metrics, including infarct volume, cerebral edema, NIH stroke score, and modified Rankin score [14, 17].

Microglia play pivotal roles in IS as the quintessential CNS resident immune cells. Microglia are a heterogeneous population, exhibiting varying functionality along spatial and temporal dimensions [18]. They can promote or suppress inflammation via multiple mechanisms but have been shown to be beneficial as a whole [19]. Efferocytosis, the phagocytosis of dead and dying cells, is well characterized as beneficial, as loss of function results in worse outcomes [20–22]. Mer tyrosine kinase (MerTK) is a phosphatidylserine recognition receptor essential for efferocytosis in microglia [23–26]. One key transcription factor that controls anti-inflammatory function in microglia and macrophages is MAF bZIP transcription factor B (MafB), a musculoaponeurotic fibrosarcoma family member [27, 28]. MafB has been shown to reduce infarct volume and neuroinflammation in tMCAO by promoting DAMP clearance in peripheral macrophages[29]. Recent transcriptomic data also revealed that MafB is crucial for late-phase tissue repair function of monocyte-derived macrophages, including myelin phagocytosis, in ischemic stroke after attenuation of early-phase microglia [30]. MafB-high microglia have been associated with brain repair and clears myelin debris [31]. MafB expression is negatively regulated by micro-RNA 155 (miR-155), a pro-inflammatory miRNA that worsens IS in rodents [32, 33]. This inverse relationship between deleterious miR-155 and protective MafB is supported by data from human IS patient serum, where miR-155 positively correlates with poor outcomes and inversely correlates with MafB mRNA levels [32, 34, 35].

We recently discovered that eCIRP causes dysfunctional bacterial phagocytosis in peripheral macrophages in the setting of sepsis [36]. Since, much of the cellular machinery of efferocytosis is shared with phagocytosis [37], we hypothesized that eCIRP released in IS, causes microglial efferocytic dysfunction and promotes brain tissue injury. We found that eCIRP is released into the cerebrospinal fluid (CSF) of tMCAO mice and that expression of efferocytic receptor MerTK is decreased in the microglia in tMCAO brains. CIRP^−/−^ tMCAO mice showed reduced neurological deficit, improved microglial MerTK expression and enhanced microglial efferocytosis of dying neurons in the peri-infarct brain relative to wild type (WT) tMCAO. As seen in WT tMCAO mice, hippocampal injection of eCIRP increased miR-155 and decreased MafB levels. In contrast, CIRP^−/−^ tMCAO mice had attenuated miR-155, but preserved MafB expression in the brain compared to WT tMCAO. Next, we employed *in vitro* primary microglial cell culture to unveil the underlying mechanism. Here we report that eCIRP initiates an efferocytosis-suppressing signaling pathway via TLR4 inducing pro-inflammatory miR-155 and downregulating MafB, thus reducing expression of downstream pro-efferocytic factors. Finally, we examined a therapeutic approach to target eCIRP using a small inhibitory peptide C23 to augment efferocytosis and improve outcomes in IS.

## Materials and methods

### Experimental animals

For experiments utilizing adult mice, 8 to 12-wk-old male wild-type (WT) C57BL/6 mice were purchased from Charles River Laboratories (Fairfield, NJ), and age- and weight-matched house-bred CIRP^−/−^ mice were used. CIRP^−/−^ mice with C57BL/6 background were kindly provided by Dr. Jun Fujita (Kyoto Univesity, Kyoto, Japan) [38]. Mice were allowed to acclimate for 1 week before use in experiments. Mice were housed at the Center for Comparative Physiology at the Feinstein Institutes for Medical Research and in a temperature-controlled room under 12 h light/12 h dark cycles and were fed standard chow diet ad libitum. For primary microglia isolation, breeding triads were closely monitored to precisely record the pups date of birth. House-bred C57BL/6 and TLR4 deficient (TLR4^−/−^) neonatal mouse pups aged 0–3 days (P0-P3) were utilized for primary microglia isolation. TLR4^−/−^ mice on the C57BL/6 background were obtained from Dr. Kevin Tracey (The Feinstein Institutes for Medical Research, Manhasset, NY). All experimental procedures were performed in accordance with the National Institutes of Health Guidelines for the Care and Use of Laboratory Animals. This study was approved by the Institutional Animal Care and Use Committee of the Feinstein Institute for Medical Research.

### Temporary middle cerebral artery occlusion (tMCAO) model of ischemic stroke

Male C57BL/6 and CIRP^−/−^ mice were utilized for tMCAO. tMCAO as previously described per the Longa method, adapted to mice with modifications [39]. Mice were weighed, and anesthesia was induced with 4% isoflurane, and maintained at 1.5–2%. Body temperature was maintained at 37° C with a heating pad throughout the procedure. Mice were placed supine, the anterior neck fur was shaved, and residual fur removed with hair removal cream. The area was disinfected, and through a 1 cm midline incision the tissues were dissected to expose the left carotid bifurcation. The vagus nerve was identified, and care was taken to avoid injury to the structure. The proximal common carotid artery (CCA) was ligated, the distal external carotid artery (ECA) was ligated tightly, and the internal carotid artery (ICA) was ligated. A proximal ECA arteriotomy was performed, and a silicone-tipped occluding suture (Doccol) was introduced and secured with a second tie on the ECA. The ICA was untied, and the occluding suture was advanced 8–10 mm to the origin of the middle cerebral artery (MCA) and fixed with silk ties on the ECA and ICA. The neck was closed with silk suture, and anesthesia withdrawn to allow for 1 hour of awake ischemic time. The animal was anesthetized, and skin prepped in the same fashion, the occluding suture was slowly withdrawn, the ECA ligated proximally, and the CCA was untied to allow for reperfusion. Hemostasis was confirmed and cervical wound closed. Mice were randomly assigned to receive vehicle (Phosphate buffered Saline, PBS) or C23 (GenScript, Piscataway, NJ). Immediately after wound closure, mice were retro-orbitally injected with 0.1 mL vehicle or C23 (8 μg/g b.w.) dissolved in PBS. Sham mice underwent the same surgical procedures, but without intraluminal suture insertion. Animals were sacrificed 24 h after reperfusion, and tissues were collected.

### CSF collection and CIRP enzyme-linked immunosorbent assay (ELISA)

CSF was collected from mice 24 h after tMCAO or sham surgery, as described previously [40]. Briefly, mice were anesthetized with 4% isoflurane, shaved and placed in a stereotaxic frame (David Kopf Instruments), with gas anesthesia nosepiece attachment. A 1 cm long incision was made from the back of the head to the nape; muscles were retracted to expose the cisterna magna membrane with care to avoid injury and bleeding. Using a micromanipulator, a pulled glass capillary was used to carefully puncture the membrane overlying the cisterna magna and CSF was allowed to flow into the capillary for 5 minutes. At the end of CSF collection, mice were sacrificed. CSF was carefully inspected for blood contamination during collection and prior to transfer into sterile tubes, only pure CSF was flash frozen in liquid nitrogen, stored at −80° C, and used for experiments. Due to varying volumes of CSF collected, sample volumes were recorded immediately prior to beginning the ELISA protocol. Mouse CIRP ELISA kit (FineTest, catalog no. EM6504), was used for quantifying CSF eCIRP concentration as per manufacturer’s protocol.

### eCIRP and peptide C23

Recombinant mouse CIRP (referred to as eCIRP) was prepared in-house, with quality control measures performed as previously described [8]. C23 (GRGFSRGGGDRGYGG) was synthesized by GenScript USA Inc. (Piscataway, NJ), purified to > 95% by HPLC, and provided as a lyophilized powder. The peptide was resuspended in sterile PBS at desired concentration prior to treatment of cells or mice.

### Stereotactic hippocampal injection

Male C57BL/6 mice were used for hippocampal injection and subjected to dorsal intra-hippocampal injection per previously published protocol [41]. Briefly, anesthesia was induced with 4% isoflurane and maintained at 1–2%. The scalp was shaved, residual fur removed with hair removal cream, the area was disinfected, and the animal was placed in the stereotactic apparatus (David Kopf Instruments). A 1cm scalp incision was made and Bregma identified at the intersection of the bregmatic and sagittal sutures. Using a stereotactically mounted Dremel, a small burr hole was drilled in the skull at AP −2.0 mm, ML, 1.5 mm. 2 μL of eCIRP or PBS was loaded into microliter syringe (Hamilton) fitted with 33-gauge needle (Hamilton). 1.17 μL (1 μg) of eCIRP was injected using the Quintessential Stereotaxic Injector (Stoelting) at a rate of 0.2 μL per min at coordinates AP −2.0 mm, ML, + 1.5 mm, DL −1.5 mm. 5 min after completion of the injection, the syringe was withdrawn, hemostasis was confirmed, and the skin was closed with silk suture. Animals were sacrificed 24 h post-operatively via cervical dislocation and both ipsilateral and contralateral hippocampal tissues were collected and frozen in liquid nitrogen before storage at −80° C.

### Bederson score for sensorimotor deficit

Mice were scored 24 h after tMCAO using a 0–5 point modified version of the originally published Bederson score system as described previously [42]. No observable deficit is scored 0, forelimb flexion on tail raise is scored 1, decreased resistance to lateral push in addition to forelimb flexion is awarded a score of 2, circling behavior is scored 3, circling and spinning or “log rolling” around the cranial-caudal axis is scored 4, and lack of spontaneous movement is scored 5.

### Triphenyltetrazolium chloride (TTC) staining

Mice were sacrificed 24 h after tMCAO, brains were removed and cut into 2 mm thick coronal sections using a stainless-steel brain matrix (Stoelting). Sections were stained at room temperature for 20 min in a 2% TTC solution in PBS, and fixed in 4% Paraformaldehyde (PFA). Photographs were taken, and edema-corrected infarct volume was determined in a blinded manner using NIH ImageJ using a previously published method for quantification and edema correction [43].

### Tissue immunofluorescence staining and Terminal deoxynucleotidyl transferase dUTP nick end labeling (TUNEL) labeling

24 h after tMCAO, mice were anesthetized deeply with 5% isoflurane and transcardially perfused with pre-perfusion buffer containing 0.5% sodium nitrite, 0.9% sodium chloride and 0.1% heparin, followed by fresh 4% PFA. Brains were harvested and post-fixed overnight at room temperature in 4% PFA and transferred the next day to 30% sucrose solution in PBS for 1–3 days. Brains were sliced into 40 μM sections on a freezing microtome. Sections were stained free floating. For efferocytosis experiments, tissues were permeabilized with 0.5% Triton X-100 in PBS at 80°C for 15 min [44]. For other immunofluorescence experiments, tissues were permeabilized using 0.2% Triton X-100 in 3% BSA in PBS. Tissues were blocked in 3% BSA in PBS for 1h. Primary antibody staining was carried out at 4°C overnight, using rabbit anti-mouse Ionized Calcium-binding Adapted Molecule 1 (Iba-1) (1:500; Wako Chemical, catalog no. 019–19741), and mouse anti-mouse Neuronal Nuclei (NeuN) (1:250; EMD Millipore, catalog no. MAB377). For MerTK quantification, primary antibody staining was conducted in the same manner using rabbit anti-mouse MerTK (1:200; Invitrogen, catalog no. 14-5751-82) and goat anti-mouse Iba-1 (1:500; Novus Biologicals, catalog no. NB100–1028). Sections were washed with 0.2% Tween-20 PBS and subsequently incubated with secondary antibodies for 1 h at room temperature with Alexa Fluor 532 goat anti-mouse (1:500; Invitrogen, catalog no. A11002) and Alexa Fluor 647 goat anti-rabbit (1:500; Invitrogen, catalog no. A21244) for efferocytosis and Alexa Fluor 594 donkey anti-rabbit (1:500; Invitrogen, catalog no. A21207) and Alexa Fluor 647 donkey anti-goat (1:500; Invitrogen, catalog no. A212447) for MerTK quantification. For efferocytosis, TUNEL staining was conducted using *In situ* cell death detection kit, Fluoroscein (Roche Diagnostics, catalog no. 11684795910), adapted for free floating samples. Free floating sections were stained in microcentrifuge tubes, with a 1:9 mixture of Tdt enzyme to labeling solution at 37°C for 1 h. Tissues were then washed with 0.2% Tween 20 in PBS, stained with 4’,6-diamidino-2-phenylindole (DAPI), mounted onto gelatin subbed slides, and coverslipped in ProlonGold antifade mountant (Invitrogen, catalog no. P36934).

### Confocal microscopy and image analysis

Brain tissues were imaged on a Zeiss LSM880 scanning confocal microscope (Carl Zeiss Imaging, Germany). The infarct region was defined as strongly positive TUNEL staining with a loss of NeuN staining, and the peri-infarct region was identified as a circumscribing area 100–150 μM around the infarct. Z-stack images were obtained in the peri-infarct cortex using a 63x objective for the full thickness of the tissue section. For each brain sample, 3 images of 2–3 different tissue sections were obtained and analyzed. Efferocytosis quantification was carried out in Imaris (Bitplane) software. Briefly, surfaces were created using Iba-1 staining for microglia and NeuN/TUNEL double positive staining for dead/dying neurons. Efferocytosed neuron surfaces were quantified using volume overlap classifier and calculated by dividing the number of completely engulfed TUNEL^+^ neurons by the total number of dead/dying neurons in image. For quantification of MerTK expression NIH ImageJ was utilized. Z-stack images were converted into maximum intensity projections, threshold function was used on the Iba-1 channel to create microglia ROIs, and ROIs were applied to MerTK channel to measure integrated density of MerTK staining in each ROI. The mean integrated density of MerTK fluorescence per microglia was calculated for each image, and 3 images obtained for ≥ 3 tissue sections were averaged and recorded for each animal.

### Single-cell RNA sequencing analysis

Single cell transcriptome dataset GSE227651 was processed, explored and Z-score violin plots were generated using Trailmaker^™^ https://app.trailmaker.parsebiosciences.com/; Parse Biosciences, analysis completed on 5/12/25). Unfiltered count matrices were uploaded and filtered by minimum number of transcripts per cell; 33013 for the sham samples and 26694 for the 24 h (day 1) tMCAO samples. Dying and dead cells were filtered by removing barcodes with > 8.89% mitochondrial reads. Outliers in the distribution of number of genes vs. transcripts were removed by fitting a linear regression model (p < 0.000092). Probable doublets were filtered using the scDblFinder method (threshold range: 0.46238–0.46758). Overall filtering rates after processing were 21.4% and 19.8% for sham and 24 h tMCAO respectively. Data from high-quality cells were normalized, integrated and subjected to principal-component analysis in Harmony. The Louvain method was used for clustering and Uniform Manifold Approximation and Projection embedding was applied to visualize the data. Microglia-specific genes were used to identify microglia by comparing cells in each cluster to other cells via the presto package and Wilcoxon rank-sum test. Violin plots for *Mafb, MiR155hg,* and *Mertk* were generated in the “Plots and Tables” module.

### Isolation and seeding of primary microglia

Primary microglia were isolated from C57BL/6 or TLR4^−/−^ P0-P3 neonatal mouse pups, adapted from previously described protocols [45]. Briefly, whole brains were harvested in Hank’s balanced salt solution and dissociated with Neural Tissue Dissociation Kit (P) (Miltenyi Biotec, catalog no. 130-092-628) per the manufacturer’s protocol. Mixed glial cells were suspended in complete microglial medium (Dulbecco’s Modified Eagle Medium (DMEM) containing 10% Fetal bovine serum (FBS), 100 U/mL penicillin/streptomycin, and 2 mM L-glutamine additionally supplemented with 0.5 ng/mL recombinant murine granulocyte-macrophage colony-stimulating factor (GM-CSF) (R&D Biosystems) and seeded in T-175 culture flasks. Thereafter, culture medium was changed twice weekly. Microglia were recovered from flasks at DIV 10–21 by manual shaking, centrifuged at 400g for 10 min, and resuspended in complete DMEM without GM-CSF prior to seeding in tissue culture plates and rested overnight for at least 16 h before treatment.

### Cell culture and treatments

All cells used were maintained in a humidified atmosphere at 37° C, 5% CO_2_. Mouse neuroblastoma Neuro-2a (N2a) cells were kindly provided by Dr. Phillipe Marambaud (Feinstein Institutes for Medical Research, Manhasset, NY) as described before [10]. N2a cells were cultured in 1:1 DMEM/Opti-minimum essential medium (Opti-MEM) supplemented with 10% FBS and 100 U/mL penicillin/streptomycin. N2a cells were detached with 0.25% trypsin-EDTA and seeded for experiments and passaged 1–2 times weekly. Microglia seeding density varied depending on downstream assay. For gene expression via RT-qPCR or protein expression via Western blot, microglia were seeded at a density of 3 × 10^5^ cells per well. For efferocytosis assays and MerTK expression flow cytometry experiments, microglia were seeded at a density of 1 × 10^5^ cells per well in Upcell place (Nunc). After resting overnight, media was exchanged to fresh DMEM containing PBS, eCIRP, C23, or eCIRP + C23, at doses specified in each experiment, and incubated for 20 h before lysis or use in flow cytometry.

### In vitro efferocytosis and MerTK flow cytometry assays

Microglia were treated with increasing doses of eCIRP (0.1, 1, and 2.5 μg/mL) for 20 h. Cell death was induced in N2a cells by exposure to 600 J/m^2^ 24 h prior to staining and co-incubation with microglia. After 24 h, N2a cells were detached, counted and stained with CellTrace Carboxy fluorescein succinimidyl ester (CFSE) (Invitrogen, catalog no. C34554) per manufacturer’s instructions. N2a cells were counted, resuspended to appropriate density and fed to microglia (3 × 10^5^ cells/well) at 1:3 ratio of microglia to N2a cells. After 1 h co-incubation, efferocytosis was terminated by washing with PBS, and cells detached for 15 min in cold PBS containing 1% FBS. Cells were then stained with antibodies, FITC anti mouse/human CD-11b (1:200; BioLegend, catalog no.101206) and Brilliant Violent anti-mouse F4/80 (1:20; BioLegend catalog no. 123132) and measured using BD FACSymphony flow cytometer (BD Biosciences, San Jose, CA, USA). FCS files were exported and analyzed in FlowJo 10.9.0 (BD Biosciences). Microglia were defined as CD11b^+^ and F4/80^+^ population. Median fluorescence intensity (MFI) of CFSE in the total microglia population and percentage of efferocytic microglia (microglia exhibiting CSFE fluorescence above no-N2a cell negative control threshold) were recorded.

### RNA isolation and real time quantitative PCR (RT-qPCR)

Total RNA was isolated from cells or whole brains with RNAspin Mini kit (Cytiva) and miRNA was isolated using miRNeasy micro kit (Qiagen). RNA concentration was measured via Nanodrop. Conventional cDNA synthesis was carried out with 300 ng – 1 μg of pure RNA using murine leukemia virus reverse transcriptase (Thermo Fisher Scientific, Waltham, MA). Quantitative PCR for relative mRNA expression was performed using specific primers (Table 1) and SYBR green (Applied Biosystems catalog no. 4312704), with GAPDH as endogenous control. For quantification of relative miR-155 expression, cDNA synthesis was carried out using miRCURY LNA RT Kit (Qiagen, cat no. 33940); real-time PCR reaction was conducted with miRCURY LNA SYBR Green PCR Kit (Qiagen, catalog no. 339346) with specific primers for mmu-miR-155–5p (Qiagen Geneglobe, catalog no. YP02119303) and endogenous control miRNA miR-103a-3p (Qiagen Geneglobe, catalog no. YP02119303). All real-time PCR reactions were carried out using QuantStudio3 real-time thermocycler (Applied Biosystems) and the ΔΔCt method was employed to assess relative expression levels of all targets.

### Western blotting

Cells or brain tissues were lysed in ice cold RIPA lysis buffer (10 mM Tris buffered Saline (TBS) pH 7.5, containing phenylmethylsulfonyl fluoride, Na-orthovanadate, protease and phosphatase inhibitor cocktails (Thermo fisher Scientific). Tubes were centrifuged at 12,000 rpm for 20 min at 4°C and protein containing supernatant collected in fresh tubes. Protein concentration was measured via DC protein assay (Bio-Rad, catalog no. 5000111). Equal amounts of protein extracts (15 μg/well for cell lysate, 50 μg/well for brain lysate) were loaded into NuPAGE^™^ 4–12% Bis-Tris gels (Invitrogen, Thermo Fisher Scientific), separated via electrophoresis, and wet-transferred onto nitrocellulose membranes. Membranes were blocked in0.1% casein in TBS for 1 h at room temperature and then incubated in blocking solution containing 0.1% Tween-20 overnight at 4°C with primary antibodies, rabbit anti-MafB (1:500; Bethyl labs, catalog no. A700–046), rabbit anti-mouse Phospho-Cofilin (Ser3) (1:1000; Cell Signaling Technology, catalog no. 3313S), and rabbit anti-mouse Cofilin (1:1000; Cell Signaling Technology, catalog no. 5175S). After washing and incubation with infrared dye-labeled secondary antibodies, membranes were imaged via Odyssey Clx Imaging system with Image Studio 5.2 software (Li-Cor Biosciences). After target band imaging, membranes were then incubated and bands imaged in the same manner to measure mouse anti-mouse β-actin antibody (1:10000; Sigma-Aldrich, catalog no. A5441) as endogenous loading control. Densitometric quantification was done using NIH ImageJ.

### Brain tissue ELISAs

Protein-containing brain tissue lysates from sham and tMCAO mice were generated as described for Western blotting methods. Brain IL-6 and TNFα levels were quantified using BD OptEIA^™^ Mouse IL-6 ELISA set (BD Biosciences, catalog no. 555240) and BD OptEIA^™^ Mouse TNFα ELISA set (BD Biosciences, catalog no. 558534) per manufacturer’s protocol. Data are normalized to total protein content measured in brain tissue lysate immediately prior to beginning the ELISA protocol.

### Statistical analysis

Data were analyzed using GraphPad Prism version 8.0 graphing and statistical analysis software (GraphPad Software Inc., La Jolla, CA) and presented as mean ± standard error of the mean (SEM). For two group comparisons, unpaired two-tailed Student’s t test was used. For multigroup comparisons One-way analysis of variance (ANOVA) and post-hoc Tukey’s multiple comparisons test was employed. To analyze experimental data with two independent variables we used two-way ANOVA or mixed-effects model (for data with missing values) and post-hoc Tukey’s multiple comparisons test. All data were tested for normality by the Shapiro-Wilk test. α was set at 0.05, thus comparisons with p < 0.05 were considered statistically significant differences.

## Results

### eCIRP is released into the CSF and impairs microglial efferocytosis in ischemic stroke

**Figure 1:**
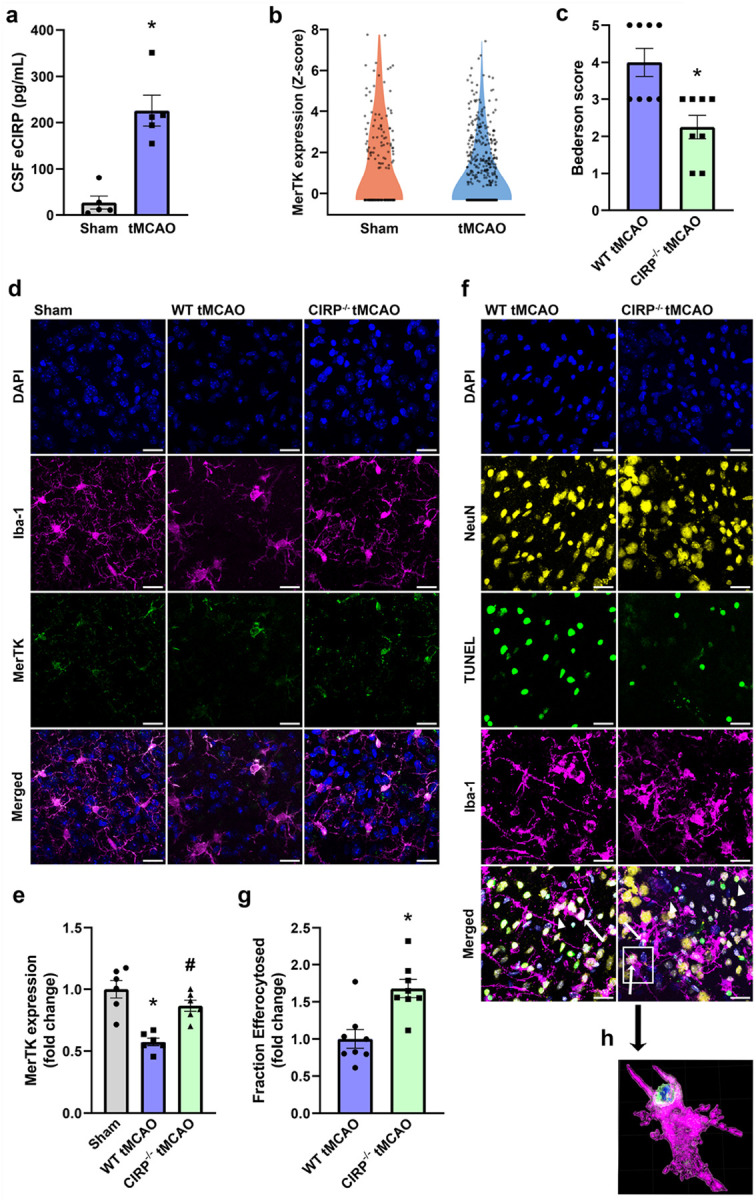
eCIRP is released in ischemic stroke and causes efferocytic dysfunction *in vivo*. **a** Concentration of eCIRP in CSF collected from mice 24 h after sham or tMCAO surgery. (*n* = 5; mean ± SEM; * *p* = 0.0006 vs. Sham; Unpaired *t*-test). **b** *Mertk* expression Z-score in microglia cluster from GSE227651. **c** Bederson score in WT tMCAO and CIRP^−/−^ tMCAO mice 24 h after surgery. (*n* = 8; mean ± SEM; * *p* = 0.0031; Unpaired *t*-test). **d** Representative confocal images of peri-infarct brain tissues in Sham, WT tMCAO, and CIRP^−/−^ tMCAO mice, stained for DAPI (blue), Iba-1 (magenta), and MerTK (green). Magnification 630x, scale bar, 20 μM. **e** Quantification of MerTK expression in microglia per animal. (*n* = 6; mean ± SEM; * *p* < 0.0001 vs. Sham; ^#^ *p* = 0.0032 vs. WT tMCAO; one-way ANOVA, Tukey’s multiple comparisons test). **f** Representative confocal images of peri-infarct brain tissues in WT tMCAO and CIRP^−/−^ tMCAO mice, with staining for nuclei (DAPI, blue), neurons (NeuN, yellow), dead/dying cells (TUNEL, green), and microglia (Iba-1, magenta); arrows indicate efferocytosis events with complete internalization of TUNEL^+^/NeuN^+^ cargo, arrowheads indicate incomplete efferocytosis events. Magnification 630x, scale bar, 20 μM. **g** Ratio of TUNEL^+^ neurons efferocytosed by microglia to total TUNEL^+^ neurons expressed as fold-change with WT tMCAO set as 1. (*n* = 8; mean ± SEM; * *p* = 0.0017 vs. WT tMCAO; Unpaired *t*-test). **h** Zoomed in high power reconstruction of 3D segmented surfaces in Imaris showing efferocytosis event indicated in white box in f, demonstrating TUNEL^+^/NeuN^+^ cargo engulfed by Iba-1^+^ microglia.

Despite eCIRP’s deleterious role in stroke [12–14], no direct measurements of eCIRP in the extracellular compartment of the brain such as CSF have been published to date. Therefore, to evaluate CSF eCIRP levels in ischemic stroke, we collected CSF from sham and tMCAO mice 24 h post-operatively and quantified CIRP via ELISA. We found eCIRP levels to be significantly elevated by 8.4-fold in the CSF of tMCAO mice compared to sham controls (Fig. 1a). To confirm that microglial efferocytosis is inefficient in early IS, we interrogated a publicly available single-cell RNA sequencing data set (GSE227651), generated from sham and tMCAO mice brains, for microglial expression of the efferocytic receptor MerTK [46]. Our analysis revealed that *Mertk* expression was reduced in microglia 24 h after tMCAO (Fig. 1b). We asked whether eCIRP could contribute to early microglial efferocytic dysfunction in IS, and to that end we subjected C57BL/6 (WT) and CIRP^−/−^ mice to tMCAO. Building on our previous study which showed that CIRP^−/−^ mice develop smaller infarcts in pMCAO[12], we assessed Bederson score, a measurement of neurological deficit, 24 h after surgery to evaluate the IS outcome. As expected, Bederson scores in CIRP^−/−^ tMCAO mice were significantly decreased by 43.8% vs. WT counterparts (Fig. 1c). Next, we measured microglial MerTK expression in WT and CIRP^−/−^ tMCAO vs. sham brains via immunofluorescence co-staining of Iba-1 and MerTK (Fig. 1d). The MerTK expression of microglia was significantly decreased by 42.6% in WT tMCAO compared to sham control brain tissue (Fig. 1d, e). CIRP deficiency rescued decreased microglial MerTK expression in tMCAO mice, which was comparable to sham in CIRP^−/−^ tMCAO brain tissue (Fig. 1d, e). Finally, we examined microglial efferocytosis of dead/dying neurons *in vivo* in tMCAO mice, via immunofluorescence staining of brain tissue sections for neuronal marker NeuN and microglial marker Iba-1, followed by TUNEL labeling to stain dead/dying cells and DAPI staining. Images were acquired from cortical peri-infarct regions, and efferocytosis was identified as engulfment and internalization of TUNEL^+^/NeuN^+^ cargo by Iba-1^+^ microglia (Fig. 1f, h). Efferocytic events were then quantified utilizing Imaris 3D segmentation software (Fig. 1h), which showed that microglial efferocytosis of TUNEL^+^ neurons was significantly increased by 1.7-fold in CIRP^−/−^ tMCAO brains compared to WT tMCAO brains (Fig. 1g). These findings show that eCIRP released in IS worsens behavioral deficit and impairs microglial efferocytic function.

### eCIRP induces pro-inflammatory miR-155 and suppresses pro-phagocytic transcription factor MafB in ischemic stroke

**Figure 2:**
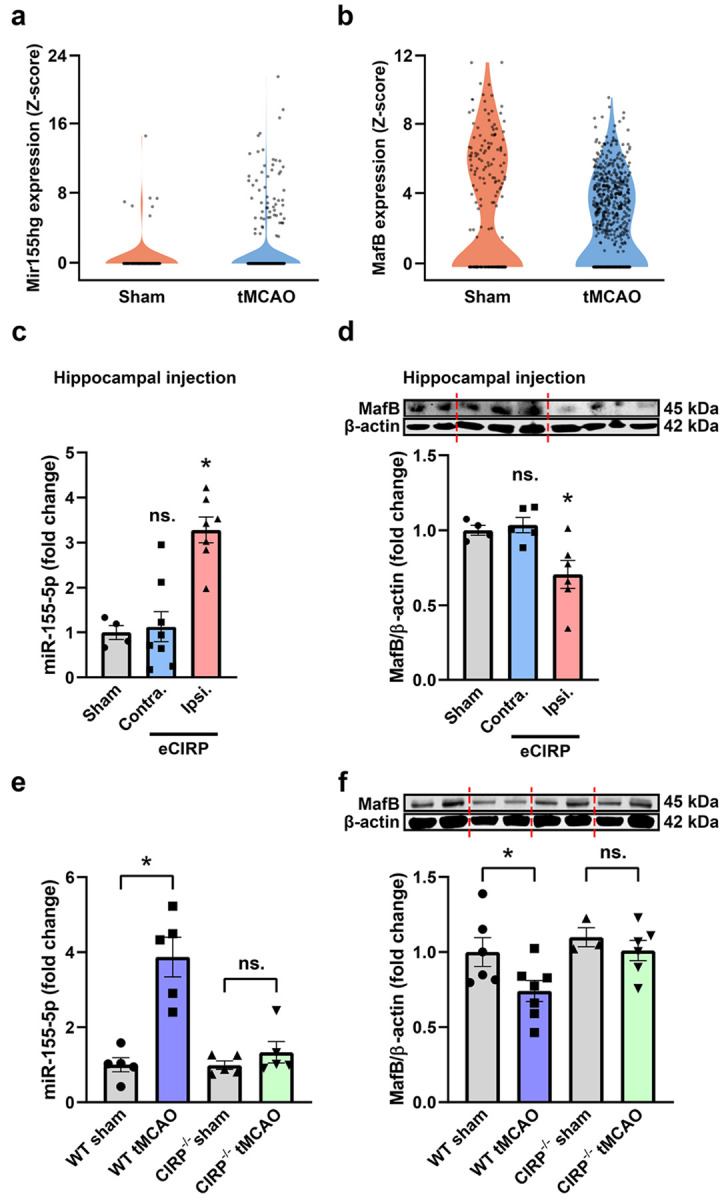
miR-155 is induced and MafB is suppressed by eCIRP in ischemic stroke. **a,b** miR-155 host gene *MiR155hg* (**a**) and *Mafb* (**b**) expression Z-scores in microglia cluster from GSE227651. **c** Relative levels of miR-155 in hippocampus 24 h after stereotactic injection of PBS or 1 μg eCIRP (Contra. & Ipsi. indicate hippocampus laterality relative to eCIRP injection). (*n* = 7–8; mean ± SEM; * *p* = 0.0009 vs. Sham & *p* = 0.0002 vs. Contra.; ns. *p* = 0.9632 vs. Sham; one-way ANOVA, Tukey’s multiple comparisons test). **d** Representative Western blot images and relative MafB protein levels in hippocampal tissues 24 h after stereotactic injection of PBS or 1 μg eCIRP (*n* = 4–6; mean ± SEM; * *p* = 0.0416 vs. Sham & *p* = 0.0159 vs. Contra.; ns. *p* = 0.946 vs. Sham; one-way ANOVA, Tukey’s multiple comparisons test). **e** Relative brain levels of miR-155 in WT and CIRP^−/−^ mice subjected to sham and tMCAO surgery. (*n* = 5; * *p* = 0.0217 WT sham vs. WT tMCAO; ns. *p* = 0.903 CIRP^−/−^ sham vs. CIRP^−/−^ tMCAO; two-way ANOVA, Tukey’s multiple comparisons test). **f** Representative Western blot images and relative MafB protein levels normalized to β-actin in whole brain lysates of WT and CIRP^−/−^ mice subjected to sham and tMCAO surgery. (*n* = 3–7; * *p* = 0.046 WT sham vs. WT tMCAO; ns. *p* = 0.7657CIRP^−/−^ sham vs. CIRP^−/−^ tMCAO; Mixed-effects model (REML) with Sidak’s multiple comparisons test).

To investigate the potential mechanism behind eCIRP’s microglial efferocytosis-inhibitory function in IS, we first checked microglial expression of *Mir155hg* and *Mafb* in single-cell RNA sequencing data set GSE227651 in tMCAO vs. sham mouse brains. *Mir155hg,* the long non-coding RNA host gene for pro-inflammatory mature miR-155, was more prevalently and highly expressed in microglia 24 h post-tMCAO versus sham control (Fig. 2a). Accordingly, the expression of *MafB,* a known target of miR-155-mediated silencing, was decreased in 24 h post-tMCAO compared to sham (Fig. 2b). To evaluate the specific role of eCIRP in regulating miR-155 and MafB without other confounding pro-inflammatory signals present in IS, we stereotactically injected eCIRP into the CA1 hippocampus region of WT mice and harvested hippocampal tissues 24 h post-injection. Injection of eCIRP increased miR-155 levels in ipsilateral (injected) hippocampal tissue by 3.3-fold, but no change was observed in contralateral (non-injected) or PBS-injected hippocampus (Fig. 2c). MafB protein levels changed inversely; ipsilateral eCIRP-injected tissues showed significant downregulation of MafB as compared to the contralateral or PBS-injected tissues (Fig. 2d). To test whether eCIRP regulates miR-155/MafB in IS, we quantified miR-155 and MafB levels in whole brain lysates collected from WT and CIRP^−/−^ mice subjected to sham or tMCAO surgery. Indeed, miR-155 increased by 3.8-fold in WT tMCAO vs. WT sham, while CIRP^−/−^ tMCAO mice showed no significant changes compared to CIRP^−/−^ sham mice (Fig. 2e). MafB levels showed a clear inverse correlation with miR-155. MafB protein decreased by 26% in WT tMCAO as compared to WT sham but not in CIRP^−/−^ tMCAO vs. CIRP^−/−^ sham (Fig. 2f). We further confirmed eCIRP-induced changes in microglial MafB expression via immunofluorescence co-staining for Iba-1 and MafB in tMCAO brain sections, which showed microglial MafB was increased by 2.2-fold in CIRP^−/−^ tMCAO compared to WT tMCAO **(Fig. S1).** Altogether, these data demonstrate that in IS, microglial miR-155 is elevated while MafB is decreased, and eCIRP alone is sufficient to mediate this pathway. Furthermore, via eCIRP loss-of-function experiments, we show that eCIRP is not only sufficient but necessary to induce miR-155 and silence MafB.

### eCIRP decreases microglia efferocytosis in vitro via the miR-155/MafB axis in a TLR4-dependent manner

**Figure 3:**
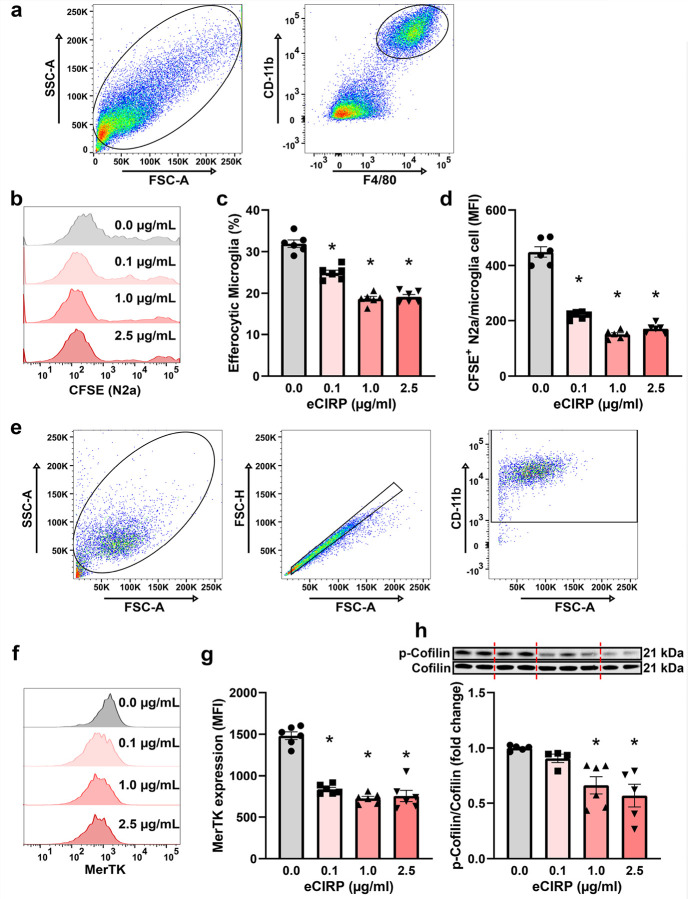
eCIRP causes microglial efferocytic dysfunction *in vitro*. **a-d** Primary microglia were stimulated with indicated eCIRP doses for 20 h and incubated with CFSE labeled dead N2a neurons at 1:3 ratio for 1 h, and efferocytosis was measured via flow cytometry. **a** Gating strategy for microglia in mixed microglia/N2a samples. **b** Representative histograms of N2a CFSE signal in CD11b^+^F4/80^+^ microglia populations at indicated doses of eCIRP. **c** Percentage of efferocytic CFSE^+^/CD-11b^+^/F4/80^+^ microglia and **d** Median fluorescence intensity (MFI) of CFSE in microglia at increasing eCIRP doses. (*n* = 6; mean ± SEM * *p* < 0.0001 vs. 0 μg/mL; one-way ANOVA, Tukey’s multiple comparisons test). **e-g** Primary microglia were stimulated with indicated eCIRP doses for 20 h, and MerTK staining was assessed via flow cytometry. **e** Gating strategy for pure microglia samples. **f** Representative histograms of MerTK signal in CD-11b^+^ gated microglia at indicated doses of eCIRP. **g** MerTK MFI of microglia at increasing eCIRP doses. (*n* = 6; mean ± SEM; * *p* < 0.0001 vs. 0 μg/mL; one-way ANOVA, Tukey’s multiple comparisons test). **h** Representative Western Blot images and quantification of p-Cofilin normalized to total Cofilin protein levels in microglia treated with indicated doses of eCIRP for 20 h. (*n* = 4–6; mean ± SEM; * *p* = 0.0156 1 μg/mL vs. 0 μg/mL & * *p* = 0.0032 2.5 μg/mL vs. 0 μg/mL; one-way ANOVA, Tukey’s multiple comparisons test).

To gain more insight into eCIRP-mediated efferocytic dysfunction, we established *in vitro* cultures of primary microglia isolated from neonatal mice. First, we conducted *in vitro* efferocytosis assays using primary microglia stimulated for 20 h with increasing concentrations of eCIRP (0, 0.1, 1, 2.5 μg/mL), followed by co-incubation with CFSE stained, UV-killed, murine Neuro-2a (N2a) neuronal cells and analyzed using flow cytometry. eCIRP significantly reduced the percentage of efferocytic microglia in a dose-dependent manner, with 1 μg/mL eCIRP decreasing the efferocytic microglia by 13.3%, a relative decrease of 41.5% (Fig. 3a–c). Importantly, eCIRP also significantly reduced the amount of N2a cells engulfed per microglia in a dose-dependent manner, with 1 μg/mL eCIRP decreasing the MFI of CFSE-stained N2a cells in microglia by 66.5% (Fig. 3d). These findings were confirmed with an imaging-based efferocytosis assay, utilizing UV-killed biotinylated N2a cells co-incubated with eCIRP-stimulated or control microglia and FITC-streptavidin staining to distinguish internalized vs. externalized efferocytosis cargo. Indeed, eCIRP-treated microglia showed a qualitative decrease in N2a efferocytosis (**Fig. S2**). We also verified eCIRP-induced MerTK downregulation *in vitro* using flow cytometry (Fig. 3e–g). eCIRP downregulated the cell surface MerTK expression in primary microglia in a dose-dependent manner, with 1 μg/mL eCIRP downregulating surface MerTK by 51.2% (Fig. 3f–g). Furthermore, *Mertk* gene expression in eCIRP stimulated microglia was also decreased by 51.8% compared to untreated controls (**Fig. S3a**). We then sought to uncover whether eCIRP has any effect on intracellular efferocytosis machinery, specifically actin binding proteins. For this, we assessed cofilin phosphorylation and Rac1 expression, previously shown to be associated with impaired bacterial phagocytosis in eCIRP-treated macrophages[36]. Much like in macrophages, microglial p-Cofilin decreased with eCIRP treatment in a dose-dependent manner, with 1 μg/mL eCIRP downregulating cofilin phosphorylation by 33.8% (Fig. 3h). Levels of Rac1 mRNA were significantly diminished by 35.8% in response to eCIRP as well (**Fig. S3b**), suggesting that eCIRP causes defects in actin dynamics necessary for efficient efferocytosis.

**Figure 4:**
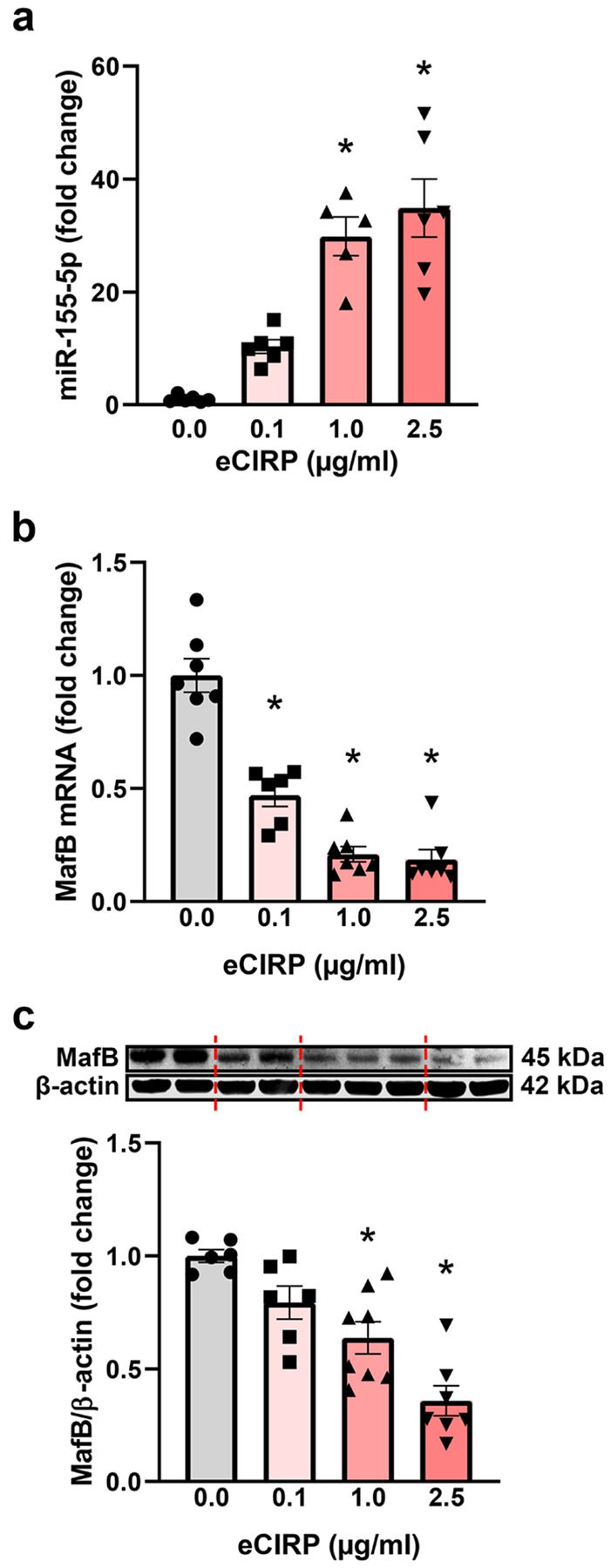
eCIRP upregulates miR-155 and downregulates MafB in primary microglia *in vitro*. Primary microglia were treated with indicated eCIRP doses for 20 h prior to RNA or protein isolation. **a** RT-qPCR quantification of relative miR-155 expression. (*n* = 5–6; mean ± SEM; * *p* < 0.0001 vs. 0 μg/mL; one-way ANOVA, Tukey’s multiple comparisons test). **b** RT-qPCR quantification of relative *Mafb* gene expression. (*n* = 6–7; mean ± SEM * *p* < 0.0001 vs. 0 μg/mL; one-way ANOVA, Tukey’s multiple comparisons test). **c** Representative Western Blot image and quantification of MafB protein expression normalized to β-actin. (*n* = 6–8; mean ± SEM; * *p* = 0.0031 1 μg/mL vs. 0 μg/mL & * *p* < 0.0001 2.5 μg/mL vs. 0 μg/mL; one-way ANOVA, Tukey’s multiple comparisons test).

**Figure 5:**
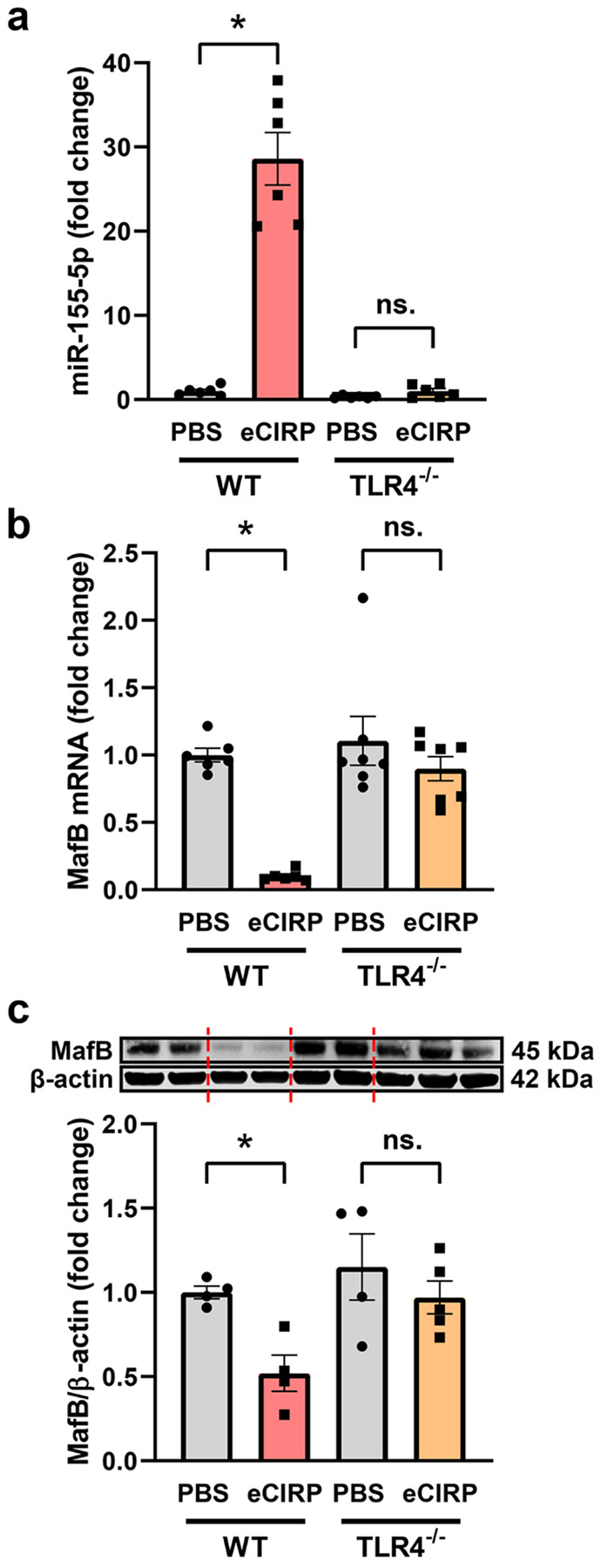
TLR4 is necessary for eCIRP-mediated regulation of miR-155 and MafB. Primary microglia were isolated from WT and TLR4^−/−^ neonatal mice and treated with PBS or 1 μg/mL eCIRP for 20 h. **a** RT-qPCR quantification of relative miR-155 expression in WT and TLR4^−/−^ microglia. (*n* = 6; mean ± SEM; * *p* = 0.0003 WT eCIRP vs. WT PBS, ns. *p* = 0.9892 TLR4^−/−^ eCIRP vs. TLR4^−/−^ PBS; two-way ANOVA, Tukey’s multiple comparisons test). **b** RT-qPCR quantification of relative *Mafb* gene expression in WT and TLR4^−/−^ microglia. (*n* = 6–7; mean ± SEM; * *p* = 0.019 WT eCIRP vs. WT PBS, ns. *p* = 0.5838 TLR4^−/−^ eCIRP vs. TLR4^−/−^ PBS; Mixed-effects model (REML) with Tukey’s multiple comparisons test). **c** Representative Western Blot images and quantification of MafB protein expression in WT and TLR4^−/−^ microglia normalized to β-actin. (*n* = 4–5; mean ± SEM; * *p* = 0.0399 WT eCIRP vs. WT PBS control, ns. *p* = 0.3991 TLR4^−/−^ eCIRP vs. TLR4^−/−^ PBS control; Mixed-effects model (REML) with Sidak’s multiple comparisons test).

Next, we verified the signaling mechanism by which eCIRP induces microglial efferocytic dysfunction using primary microglia. eCIRP increased miR-155 dose-dependently, with 29.9-fold induction at 1 μg/mL and 34.9-fold induction at 2.5 μg/mL relative to baseline (Fig. 4a). Conversely, eCIRP significantly downregulated MafB mRNA levels in a dose-dependent manner, with a 77.9% decrease from the baseline expression at 1 μg/mL (Fig. 4b). MafB protein levels had a similar pattern and decreased by 36.2% and 63.3% at 1 and 2.5 μg/mL eCIRP respectively (Fig. 4c). Of note, eCIRP also diminished the mRNA expression of MafB transcriptional target C1q by 94.6% at 1 μg/mL, verifying that MafB activity was also significantly suppressed (**Fig. S4**). Additionally, BV-2 cells, an immortalized murine microglial cell line, transfected with a miR-155 mimic expressed less MafB protein relative to control mimic with levels comparable to eCIRP stimulated BV-2 cells, confirming miR-155’s role in translational repression of MafB (**Fig. S5**). Since eCIRP is known to bind to TLR4 and induction of miR-155 in brain has been shown to be TLR4-dependent [47], we examined whether TLR4 was required to initiate miR-155 expression and resultant MafB suppression in microglia. Therefore, we isolated primary microglia from WT and TLR4^−/−^ neonatal mice and quantified miR-155, MafB mRNA, and MafB protein levels. In contrast to WT microglia, TLR4^−/−^ microglia did not significantly upregulate miR-155 when stimulated with eCIRP (Fig. 5a). Accordingly, unlike WT microglia TLR4^−/−^ microglia also did not downregulate MafB mRNA (Fig. 5b) or MafB protein levels (Fig. 5c). These data collectively show that eCIRP induces microglial efferocytosis dysfunction via TLR4/miR-155/MafB axis.

### Targeted inhibition of eCIRP/TLR4 binding with small peptide C23 rescues efferocytic dysfunction in ischemic stroke

**Figure 6:**
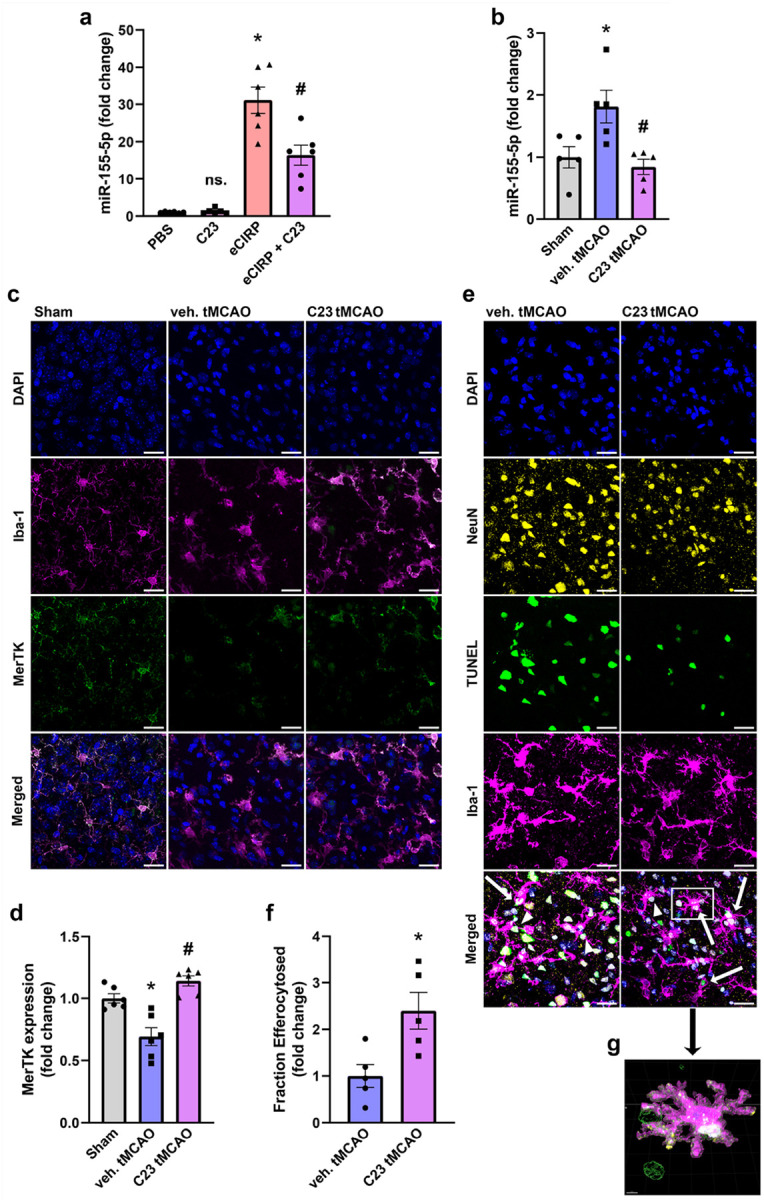
C23, a small peptide inhibitor of eCIRP/TLR4 binding, attenuates miR-155 induction *in vitro* and *in vivo* and rescues MerTK expression and microglial efferocytosis in ischemic stroke. **a** Primary microglia were treated with C23 (1 μg/mL), eCIRP (1 μg/mL), or a mixture of eCIRP and C23 for 20 h and miR-155 expression was quantified via RT-qPCR. (*n* = 6; mean ± SEM; * *p* < 0.0001 vs. PBS, ^#^ *p* = 0.0008 eCIRP + C23 vs. eCIRP; one-way ANOVA, Tukey’s multiple comparisons test). **b-g** WT mice were subjected to sham or tMCAO surgery and received retroorbital injection of C23 or vehicle solution (veh., PBS). **b** RT-qPCR quantification of relative miR-155 levels in whole brain lysates of Sham, veh. tMCAO, and C23 tMCAO mice. (*n* = 5; mean ± SEM; * *p* = 0.0298 veh. tMCaO vs. Sham; ^#^ *p* = 0.0109 C23 tMCAO vs. veh. tMCAO; one-way ANOVA, Tukey’s multiple comparisons test). **c** Representative confocal images of peri-infarct brain tissues in Sham, veh. tMCAO and C23 tMCAO mice, stained for DAPI (blue), Iba-1 (magenta), and MerTK (green). Magnification 630x, scale bar, 20 μM. **d** Quantification of MerTK expression in microglia. (*n* = 6; mean ± SEM; * *p* = 0.0022 veh. tMCAO vs. Sham; ^#^ *p* < 0.0001 C23 tMCAO vs. veh. tMCAO; one-way ANOVA, Tukey’s multiple comparisons test). **e** Representative confocal images of peri-infarct brain tissues in veh. tMCAO and C23 tMCAO mice, with staining for nuclei (DAPI, blue), neurons (NeuN, yellow), dead/dying cells (TUNEL, green), and microglia (Iba-1, magenta); arrows indicate efferocytosis events with complete internalization of TUNEL^+^/NeuN^+^ cargo, arrowheads indicate incomplete efferocytosis events. Magnification 630x, scale bar, 20 μM. **f** Ratio of TUNEL^+^ neurons completely engulfed by microglia to total TUNEL^+^ neurons expressed as fold-change with veh. tMCAO set as 1. (*n* = 5; mean ± SEM; * *p* = 0.0168; Unpaired *t*-test). **g** Zoomed in high power reconstruction of 3D segmented surfaces in Imaris showing efferocytosis event indicated in white box in **e**, demonstrating TUNEL^+^/NeuN^+^ cargo engulfed by Iba-1^+^ microglia.

After characterizing the mechanism of eCIRP-mediated microglial efferocytic dysfunction, we tested a therapeutic approach to antagonize eCIRP/TLR4 interaction. First, we treated microglia with C23, eCIRP, or a mixture of eCIRP and C23 (eCIRP + C23) and compared miR-155 expression across these groups. C23 alone did not affect basal miR-155 levels of primary microglia and as expected eCIRP induced miR-155 expression by 31.2-fold (Fig. 6a). Indeed, co-treatment with C23 and eCIRP attenuated eCIRP-induced miR-155 expression by 47.3% (Fig. 6a). To test C23’s effect *in vivo,* mice were subjected to tMCAO and retro-orbitally injected at time of reperfusion with C23 (10 μg/g b.w.) or PBS vehicle solution (veh.). Brain levels of miR-155 were increased by 1.8-fold in veh. tMCAO mice compared to sham mice, while C23 treatment significantly attenuated the miR-155 levels in tMCAO mice bringing them down to sham levels, (Fig. 6b). Microglial MerTK expression measured by immunofluorescence co-staining with Iba-1 and MerTK (Fig. 6c), demonstrated a 30.7% decrease in MerTK expression in veh. tMCAO brain tissue with respect to sham control (Fig. 6d). However, C23 treatment completely abrogated this reduction in MerTK expression (Fig. 6c, d). Subsequently, we assessed efferocytosis in peri-infarct regions of tMCAO brains via staining for Iba-1, NeuN, and DAPI, followed by TUNEL labeling (Fig. 6e), and 3D-segmentation analysis in Imaris (Fig. 6g), which showed a 2.4-fold increase in microglial efferocytosis in the C23 treated group compared to vehicle treated tMCAO (Fig. 6f). Thus, C23 attenuates eCIRP-mediated miR-155 induction *in vitro* and *in vivo*, and improves microglial expression of MerTK and rescues microglial efferocytic dysfunction in IS.

### C23 treatment improves ischemic stroke outcome

**Figure 7:**
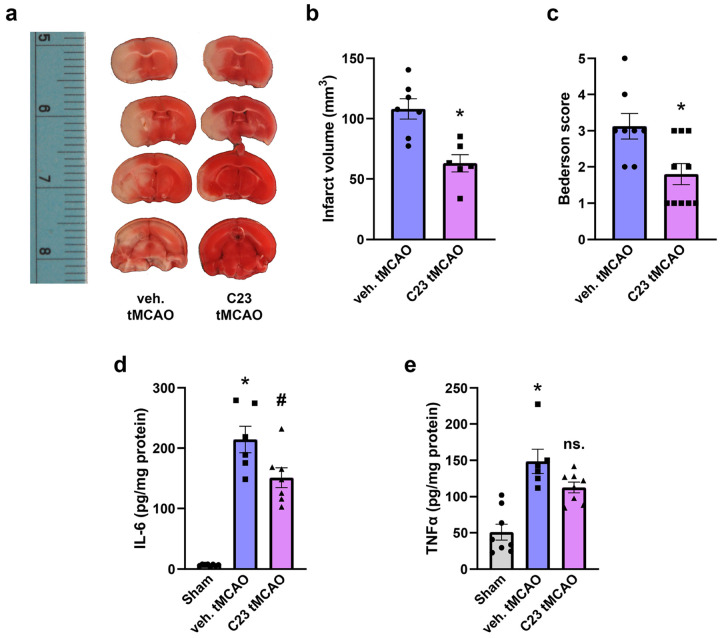
C23 treatment improves outcomes in murine ischemic stroke. WT mice were subjected to Sham or tMCAO surgery and received retroorbital injection of C23 or vehicle solution (veh., PBS). **a** Representative images of 2 mm thick brain sections stained with TTC for infarction detection and quantification. Infarcted brain tissue is stained pale white, while healthy brain tissue is stained in red. **b** Infarct volume in veh. or C23 treated mice 24 h after tMCAO quantified with NIH ImageJ. (*n* = 6–7; mean ± SEM; * *p* = 0.0021; Unpaired t-test). **c** Bederson score 24 h after tMCAO in veh. vs. C23 treated mice (*n* = 8–10; mean ± SEM; * *p* = 0.0097; Unpaired t-test). **d-e** 24 h post-reperfusion, whole brains were collected and protein isolated. **d** IL-6 tissue ELISA expressed as pg IL-6/mg total protein. (*n* = 6–8; * *p* < 0.0001 vs. Sham; ^#^ *p* = 0.02 vs. veh. tMCAO; one-way ANOVA, Tukey’s multiple comparisons test). **e** TNFα tissue ELISA expressed as pg TNFα/mg total protein. (*n* = 6–8; * *p* < 0.0001 vs. Sham; ns. *p* = 0.1056 vs. veh. tMCAO; one-way ANOVA, Tukey’s multiple comparisons test).

We then sought to evaluate the efficacy of C23 in improving outcome measurements as a neuroprotective and immunomodulatory agent in IS. We evaluated acute outcomes 24 h post-tMCAO after a one-time dose of C23 injected retro-orbitally. Triphenyl tetrazolium chloride (TTC) staining of fresh brain sections was conducted exhibiting a decrease in infarction in tMCAO mice that received C23 compared to mice treated with vehicle (Fig. 7a). Quantification of the infarct volume showed a significant reduction by 41.7% in C23 treated tMCAO mice compared to veh. treated tMCAO mice (Fig. 7b). C23 was also successful in reducing sensorimotor deficit with C23 treated mice exhibiting forelimb flexion and decreased resistance to lateral push while vehicle treated mice exhibited unidirectional circling, shown as 42.3% decrease in Bederson score in the C23 tMCAO mice (Fig. 7c). In addition, C23 significantly attenuated brain levels of IL-6 by 29.4% vs. veh. treatment (Fig, 7d), and TNFα levels tended to decrease compared to veh. tMCAO (Fig. 7e). Thus, our findings show that C23 is a novel neuroprotective therapy that limits infarction and behavioral deficit in ischemic stroke, which warrants further evaluation.

## Discussion

The emerging role of eCIRP, in post-stroke neuroinflammation is intriguing and needs to be completely elucidated. Efferocytosis is a relatively new area of study that is garnering increasing attention in the field of IS pathology and drug development [22, 48, 49]. Microglia and infiltrating peripheral macrophages have been shown to conduct efferocytosis in IS [48, 50, 51]. eCIRP activates microglia and shifts microglia to a “classical” pro-inflammatory phenotype via NF-κB [12, 15]. However, it remains to be determined whether eCIRP regulates microglial efferocytosis in IS, if so, what specific molecular mechanisms are involved. Discovery of molecular targets of eCIRP associated with microglial efferocytosis could lead to novel targeted therapies capable of enhancing efferocytosis in IS.

**Figure 8:**
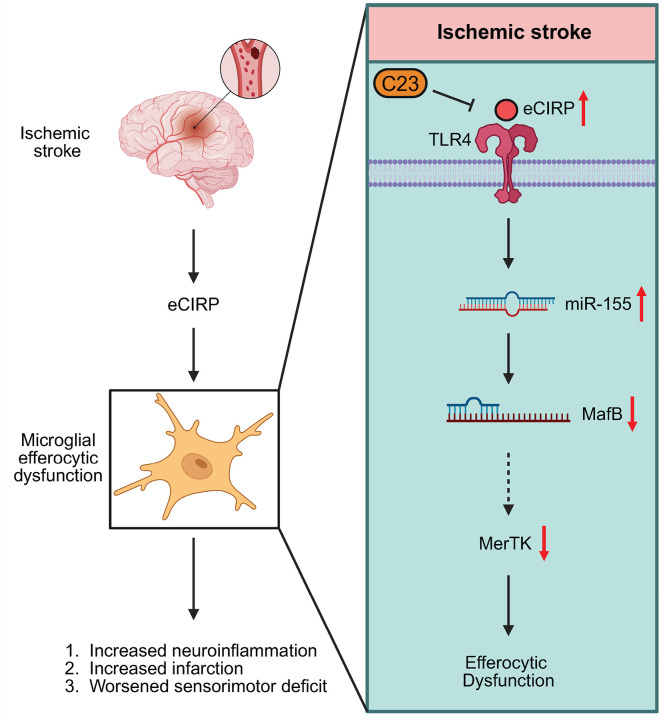
Schematic diagram summary. eCIRP is released in stroke brain and binds to TLR4 expressed on microglia and induces miR-155 and suppresses MafB, causing decreased MerTK expression and impaired efferocytosis. In addition, inhibition of eCIRP-TLR4 interaction with small peptide C23 attenuates eCIRP-induced microglial miR-155 expression and downstream microglial efferocytic dysfunction.

Herein, we report that eCIRP via TLR4 binding regulates the miR-155/MafB pathway to downregulate MerTK and induce microglial efferocytic dysfunction in IS and targeting this interaction with small inhibitory peptide C23 improves IS outcomes (Fig. 8). First utilizing the tMCAO model of IS, we showed that eCIRP is released into CSF and microglial MerTK expression is decreased *in vivo* 24 h post-stroke. Next, using tMCAO in CIRP-deficient mice we showed that eCIRP worsens the IS outcome, diminishes MerTK expression and impairs microglial efferocytosis function. We then illustrated that microglial miR-155 expression was increased, while microglial MafB expression was reduced 24 h post-tMCAO. This inverse association in miR-155 and MafB expression was then recapitulated at the site of hippocampal eCIRP injection. In contrast, CIRP^−/−^ tMCAO mice had attenuated miR-155, but preserved MafB expression in the brain compared to WT counterparts. We further investigated this mechanism *in vitro* showing that eCIRP regulated microglial efferocytosis via the miR-155/MafB axis in a TLR4-dependent manner. Furthermore, we demonstrated that C23, a small peptide blocking eCIRP-TLR4 interaction, rescued microglial efferocytic dysfunction in tMCAO by attenuating miR-155 and increasing MerTK. C23 reduced levels of inflammatory cytokines, infarct volume and neurological impairment in tMCAO indicating improved stroke outcome.

The role of miR-155 is widely studied and this miRNA is known to be induced in inflammation, and silence MafB translation [32]. Our work demonstrates that eCIRP is a potent inducer of miR-155 *in vitro* and *in vivo*, and furthermore suggests that eCIRP may be the main inducer in tMCAO. eCIRP precipitously and dose-dependently decreased microglial MafB at the mRNA and protein levels *in vitro*. MafB was also downregulated by eCIRP *in vivo*. We show that eCIRP’s effects on miR-155 and MafB are dependent on microglial expression of TLR4 corroborating previous work on miR-155 which demonstrated that TLR4 is necessary for miR-155 induction in the brain [47, 52]. The literature clearly supports that loss of function or early inhibition of miR-155 improves IS outcomes [32, 33, 52–54]. In contrast, distal MCAO mice treated with a miR-155 inhibitor 48 h post-stroke exhibited decreased expression of CD68, a phagocytic marker, in peri-vascular microglia and macrophages [55]. Together, these data suggest that miR-155 is an important inducer of post-stroke inflammation which amplifies tissue-degenerative early neuroinflammation, as we demonstrate here, but may also play a role in later-phase tissue restorative neuroinflammation. Accordingly, miR-155’s target, MafB is known to be anti-inflammatory and pro-phagocytic. MafB is well characterized to promote phagocytosis and efferocytosis in peripheral macrophage cell lines [56, 57]. MafB has also been shown to augment DAMP scavenging by infiltrating peripheral macrophages in IS [29]. Furthermore, activation of MafB with a synthetic retinoid reduced infarct volume and neurological deficit from 4–28 d post injury supporting its tissue-restorative role [29]. Broadly, MafB is emerging as a critical transcription factor for adopting the tissue-reparative phenotype in microglia and macrophages [30, 31]. Hence, eCIRP-mediated MafB silencing is likely to impair the tissue-restorative functions of microglia and macrophages beyond its impact on efferocytosis.

Of note, we found that eCIRP dramatically suppressed C1q, a transcriptional target of MafB [58]. Canonically, C1q is the initiator of the classical complement pathway functioning to opsonize or lyse exogenous pathogens [59]. However recently, C1q has been shown to augment efferocytosis, promote MerTK expression, and inhibit pro-inflammatory cytokine secretion in macrophages [60–63]. This mechanism is not yet well understood but an AMPKα-mediated mechanism has been proposed [62]. Taken together, these previous findings and our present data suggest that C1q suppression is likely to be a key factor in microglial efferocytic dysfunction. Though C1q could be involved based on current findings, considering diverse roles of C1q in ischemic stroke settings, further detailed studies will be needed which are beyond the scope of this project.

In the difficult landscape of IS drug development, precisely targeted therapeutic agents are a promising avenue. Immunosuppressive corticosteroid therapy is not recommended for IS, due to increased risk of opportunistic infections in the setting of post-stroke immunodepression [64, 65]. Therefore, targeting specific pathways that promote tissue restoration and dampen exaggerated neuroinflammation are necessary. We demonstrated that C23 abrogates miR-155 induction and enhances microglial efferocytosis, improving the stroke outcome measurements of infarct volume and sensorimotor function. Recently, another group has reported that C23 improved blood-brain-barrier integrity in mice subjected to tMCAO [14]. Furthermore, a modified C23-derived peptide significantly reduced infarct volume and sensorimotor deficits in a rhesus macaque IS model [13]. Taken together, the present and recently published data conclude that C23 is a potentially efficacious peptide therapeutic for IS.

There are several limitations to our study. First, CIRP^−/−^ mice do not express the intracellular form of CIRP. Intracellular CIRP is known to be neuroprotective; however, in our extensive experience investigating eCIRP, we have observed that eCIRP’s pro-inflammatory effect is significantly more potent than any possible anti-inflammatory effect conferred by intracellular CIRP [16, 66]. Another limitation of the present work is the acute 24 h time point, which does not allow for conclusions regarding the long-term benefits of C23 treatment. We chose this timepoint to best assess acute-phase microglial efferocytosis prior to significant influx of peripheral macrophages [67]. Moreover, further investigation is needed to explore mechanistic link between MafB suppression and MerTK downregulation to clarify the intracellular pathways underlying eCIRP-mediated efferocytosis dysfunction. Finally, our translational experiments only used young male mice, therefore further translational studies including female, aged, and hypertensive or diabetic mice are needed to evaluate C23 as an IS therapeutic.

## Conclusion

In conclusion, we have demonstrated that eCIRP is a critical regulator of microglial efferocytosis via the TLR4/miR-155/MafB pathway in IS. Our translational study shows promising improvements in IS outcome measurements, demonstrating the potential utility of C23 as a novel pharmacologic therapy.

## Supplementary Files

This is a list of supplementary files associated with this preprint. Click to download.

- JNeuroinflamSupplementaryfile.docx

## Figures and Tables

**Table 1:** RT-qPCR primers used in this study.

RefSeq Accession #	Target	Forward primer (5′-3′)	Reverse primer (5′-3′)
NM_010658.3	*Mafb*	TGAATTTGCTGGCACTGCTG	AAGCACCATGCGGTTCATACA
NM_008587.3	*Mertk*	AAGGTCCCCGTCTGTCCTAA	GCGGGGAGGGGATTACTTTG
NM_007572.2	*C1qa*	AGAGGGGAGCCAGGAGC	CTTTCACGCCCTTCAGTCCT
NM_001411845.1	*GAPDH*	CATCACTGCCACCCAGAAGACTG	ATGCCAGTGAGCTTCCCGTTCAG

## Data Availability

The datasets generated during the current study are available from the corresponding author on reasonable request. The single-cell RNA sequencing dataset analyzed in this study is publicly available under accession number GSE227651 in the NCBI Gene Expression Omnibus repository.

## List of abbreviations

IS: Ischemic stroke
tMCAO: Temporary middle cerebral artery occlusion
eCIRP: Extracellular cold-inducible RNA-binding protein
DAMPs: Damage-associated molecular patterns
TLR4: Toll-like receptor 4
CIRP^−/−^: CIRP deficient
TLR4^−/−^: TLR4 deficient
pMCAO: Permanent middle cerebral artery occlusion
MerTK: Mer tyrosine kinase
MafB: MAF bZIP transcription factor B
miR: 155 micro-RNA-155
CSF: Cerebrospinal fluid
WT: Wild-type
CCA: Common carotid artery
ECA: External carotid artery
ICA: Internal carotid artery
MCA: Middle cerebral artery
TTC: Triphenyltetrazolium chloride
TUNEL: Terminal deoxynucleotidyl transferase dUTP nick end labeling
N2a: Neuro-2a
NeuN: Neuronal Nuclei
Iba-1: Ionized Calcium-binding Adapted Molecule 1
MFI: Median fluorescence intensity
Veh: Vehicle solution
ANOVA: One-way analysis of variance
SEM: Standard error of the mean
RT-qPCR: Real time quantitative PCR
ELISA: Enzyme-linked immunosorbent assay
TBS: Tris-buffered Saline
PBS: Phosphate buffered Saline
PFA: Paraformaldehyde
GM-CSF: Granulocyte-macrophage colony-stimulating factor
DMEM: Dulbecco’s Modified Eagle Medium
FBS: Fetal bovine serum
CFSE: Carboxy fluorescein succinimidyl ester
DAPI 4’: 6-Diamidino-2-phenylindole
